# Self-Assembly of Bi_2_Te_3_-Nanoplate/Graphene-Nanosheet Hybrid by One-Pot Route and Its Improved Li-Storage Properties

**DOI:** 10.3390/ma5071275

**Published:** 2012-07-23

**Authors:** Fangfang Tu, Jian Xie, Gaoshao Cao, Xinbing Zhao

**Affiliations:** State Key Laboratory of Silicon Materials, Department of Materials Science and Engineering, Zhejiang University, Hangzhou 310027, China; E-Mails: fangfangtu1990@126.com (F.T.); gscao@zju.edu.cn (G.C.); zhaoxb@zju.edu.cn (X.Z.)

**Keywords:** bismuth telluride, graphene, Li-ion batteries, sandwich

## Abstract

A sandwich structured Bi_2_Te_3_-nanoplates/graphene-nanosheet (Bi_2_Te_3_/G) hybrid has been synthesized by a facile in situ solvothermal route and has been investigated as a potential anode material for Li-ion batteries. Bi_2_Te_3_ grows during the solvothermal process with the simultaneous reduction of graphite oxide into graphene. The in situ formation process of the hybrid has been investigated by X-ray diffraction and X-ray photoelectron spectra. The Li-storage mechanism and performance of Bi_2_Te_3_/G and bare Bi_2_Te_3 _have been studied by galvanostatic cycling and cyclic voltammetry. The Bi_2_Te_3_/G sandwich exhibits an obviously improved cycling stability compared to bare Bi_2_Te_3_. The enhancement in electrochemical performance can be attributed to the combined conducting, confining and dispersing effects of graphene for Bi_2_Te_3_ nanoplates and to the self-assembled sandwich structure.

## 1. Introduction

Although carbon based materials are presently the dominant anodes in commercial Li-ion batteries, there is still a great challenge to develop alternative anode materials in order to meet the requirement of high-performance Li-ion batteries [1,2]. Alloy materials have been regarded as one of the most promising anodes for the next-generation Li-ion batteries because of their safe operating voltage and the large energy density, especially the large volumetric energy density due to the high density [3,4]. Among various elements that can form Li-alloys, Sb has received a special interest because of its appropriate alloying/de-alloying voltage (around 0.8 V *vs.* Li/Li^+^) and relatively large Li-storage capacity (660 mAh g^−1^) with the formation of Li_3_Sb composition [5]. However, the volume change is over 200% during the conversion from Sb to Li_3_Sb [6]. The volume change causes the cracking and crumbling of the particles, resulting in the loss of physical contact between the active particles and the current collector and the consequent capacity loss upon cycling.

Many measures have been taken to alleviate the volume changes for Sb during alloying/de-alloying processes. Previous work [7,8,9,10] showed that the cycling stability of metallic Sb can be improved by using Sb-based intermetallic compound MSb_x_, where M is an electrochemically inert element and acts as the buffering matrix. The improvement in electrochemical performance could also be realized by using nanostructured materials [11,12,13,14] due the large surface area, short Li-ion diffusion path and increased mechanical strength. However, the nanoparticles tend to aggregate, also leading to the disconnection of active material with the current collector. An effective approach to overcome this problem is to immobilize the nanoparticles on a matrix. Carbon-based materials [15,16,17,18] are considered as the ideal matrices since they are electrically conductive and electrochemically active in addition to the buffering effect for the volume change.

Graphene, a flat monolayer of sp^2^-bonded carbon atoms [19], was also the ideal matrix because of its appealing properties such as high electronic conductivity [20], high specific surface area [21] and high mechanical strength [22]. Our previous work showed that the cycling stability of some Sb-based intermetallic compounds could be improved by forming nanocomposites with graphene [23,24]. Similar to Sb, Bi in the same group can also electrochemically store Li by forming a Li_3_Bi composition [5,25,26,27]. So far, the effect of graphene on the electrochemical performance of Bi-based anodes has not been reported yet. Herein, an in situ solvothermal route was used to prepare the Bi_2_Te_3_-nanoplate/graphene-nanosheet (Bi_2_Te_3_/G), where Te can also reversibly store Li by forming Li_2_Te [27]. The results showed that an obvious improvement in cycling stability of Bi_2_Te_3_ could be realized by constructing a Bi_2_Te_3_/G hybrid with a sandwich structure.

## 2. Results and Discussion

**Figure 1:**
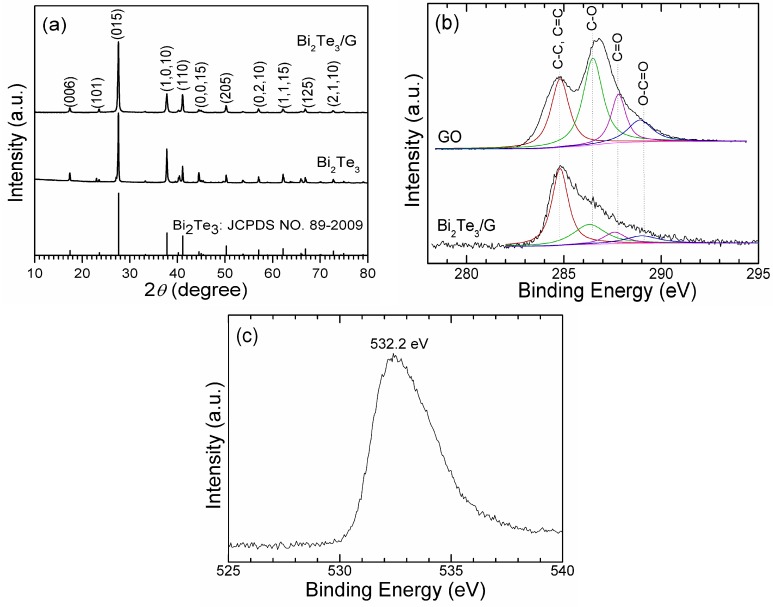
(**a**) XRD of Bi_2_Te_3_/G and bare Bi_2_Te_3_; (**b**) C1s XPS of GO and Bi_2_Te_3_/G; (**c**) O1s XPS of Bi_2_Te_3_/G.

Figure 1a shows the XRD patterns of Bi _2_Te_3_/G and bare Bi_2_Te_3_. For comparison, the standard diffraction peaks of Bi_2_Te_3_ are also given. All the diffraction peaks can be indexed to hexagonal Bi_2_Te_3_ (JCPDS No. 89-2009) for both Bi_2_Te_3_/G and bare Bi_2_Te_3_. The diffraction peaks of graphene that should appear at around 2*θ* = 25 °C cannot be detected, suggesting that the restacking of graphene sheets after reduction was inhibited by uniformly loading Bi_2_Te_3_ plates between the graphene sheets. The graphene content is roughly estimated to be 11.5 wt% by the carbon content analysis.

To check the reduction status of GO during the solvothermal reactions, C1s XPS spectra of GO and Bi_2_Te_3_/G are analyzed as shown in Figure 1b. The spectra can be fitted by four peaks for different forms of carbons: non-oxygenated carbon (C-C, 285.6 eV and C=C, 284.8 eV), carbon in C-O bonds (286.3 eV), carbonyl carbon (C=O, 287.6 eV) and carboxylate carbon (O-C=O, 289.0 eV) [28]. After the solvothermal reactions, the peak intensity of the oxygenated carbons (C-O, C=O, O-C=O) shows an obvious decrease, indicating the reduction of GO into graphene. Figure 1c gives the O1s XPS spectra of the hybrid. The broad peak at 532.2 eV is assigned to the residual oxygen-containing groups such as -COOH and -OH [29], which agrees with the result of C1s spectra. From the XRD and XPS analyses, it can be concluded that a Bi_2_Te_3_/G hybrid has formed with the simultaneous formation Bi_2_Te_3_ and reduction of GO during the one-step solvothermal process. 

**Figure 2:**
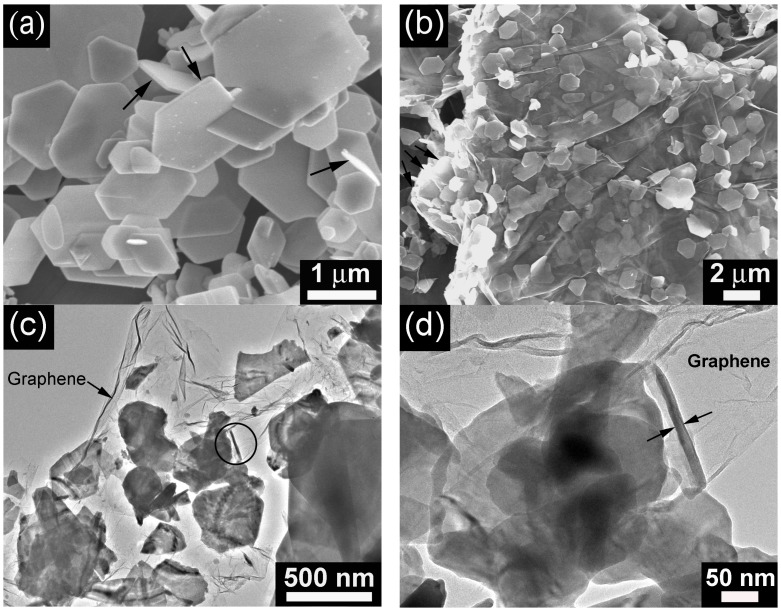
(**a**) SEM image of bare Bi_2_Te_3_; (**b**) SEM image of Bi_2_Te_3_/G hybrid; (**c**) TEM image of Bi_2_Te_3_/G hybrid; (**d**) the enlarged view of the circled part in (**c**).

Figure 2a shows the SEM image of bare Bi _2_Te_3_ prepared by the solvothermal route without adding GO in the precursors. Generally, the sample exhibits a hexagonal plate shape with a size of around 0.5–3 μm. However, smaller sized broken plates with an irregular shape can also be observed. The thickness of the plates is roughly estimated to be 50–100 nm from some vertically (or partially vertically) aligned plates denoted by the arrows. Figure 2b shows the SEM image of the Bi_2_Te_3_/G hybrid. It is obvious from the transparent graphene that Bi_2_Te_3 _plates are located between the graphene sheets. The size of the Bi_2_Te_3 _plates is below 2 μm. A three-dimensional (3D) sandwich structure is thus constructed by alternatively arranged graphene layer and Bi_2_Te_3_ layer as clearly indicated by the arrows. The transparent nature of the graphene layer suggests that it is rather thin, composed probably of single or few layers of graphene sheets.

The microstructure of Bi_2_Te_3_/G was further characterized by TEM as shown in 2c. Note that nearly all of the Bi _2_Te_3_ plates are anchoring on graphene without forming free Bi_2_Te_3_ plates even after vigorous ultrasonication. The wrinkles and folded edges of graphene indicate that the graphene layer is rather thin, which agrees with the SEM observation. The enlarged view of the circled part in Figure 2c is presented in Figure 2d. The overlapping of the Bi_2_Te_3_ plates can be observed from Figure 2d, from which the transparent nature of the Bi_2_Te_3_ plate is evident. From a vertically aligned plate, the thickness of the plate is estimated to be 15 nm, which is much smaller compared with that of bare Bi_2_Te_3_. The thickness of the Bi_2_Te_3_ plates is in the range of 10−20 nm after observing some plates at the edges or defect sites of graphene. It is worth noting that the number of the Bi_2_Te_3_ plates with a regular hexagon is much smaller compared with that of bare Bi_2_Te_3_ plates. It is believed that the irregular shape and small thickness of the Bi_2_Te_3_ plates in Bi_2_Te_3_/G are caused by the inhibited diffusion and re-crystallization of small sized Bi_2_Te_3 _particles due to the pinning effect of the defects and the oxygen-containing groups in graphene [30]. The presence of the residual oxygen-containing groups can be confirmed by XPS shown in Figure 1b,c.

**Figure 3:**
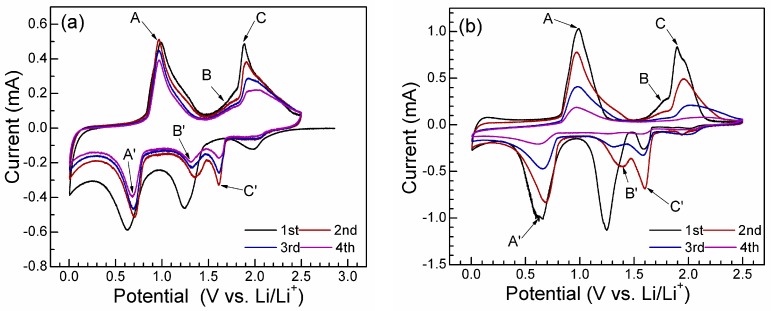
CV plots of (**a**) Bi_2_Te_3_/G; and (**b**) Bi_2_Te_3 _scanned at 0.1 mV s^−1^.

In Figure 3, CV plots of Bi_2_Te_3_/G and Bi_2_Te_3_ were measured to clarify the electrochemical reaction mechanism of Bi_2_Te_3_ with Li. As seen in Figure 3a the reaction Bi _2_Te_3 _with Li exhibits a multi-step mechanism, evidenced by the multi-peak feature of the CV plots. During the first cathodic scan, three reduction peaks, located at 2.0, 1.25, and 0.6 V, respectively, appear as indicated by the black line in 3a. The small peak at 2.0 V is related possibly to the insertion reaction of Li ion into layered Bi _2_Te_3 _without significantly destroying its crystal structure [31]. The peak at 1.2 V corresponds to the lithiation reaction of Te by forming Li_2_Te (2Li + Te → Li_2_Te) [27], and the peak at 0.6 V is associated with the reaction of Bi with Li to form Li_3_Bi (3Li + Bi → Li_3_Bi) [5,26,27]. During the second cathodic scan, the peak at 2.0 V almost disappears, the peak at 0.6 V is shifted to a higher value of 0.7 V (A’), while the peak at 1.2 V is divided into two peaks, B’ and C’, located at 1.35 and 1.65 V, respectively. The division of the peak means that the reaction of Te with Li occurs via a two-step mechanism by forming LiTe_3 _and Li_2_Te successively, both of which are stable phases at room temperature [32]. The lower peak potential in the first cathodic scan compared with those in the subsequent scans is due to the additional energy required for the displacement reactions of Bi_2_Te_3 _upon Li uptake [4]. This also means that structure of Bi_2_Te_3_ cannot be recovered after the anodic scan [27].

Note that three oxidation peaks (A, B, and C), at 1.0, 1.8 and 1.9 V, respectively, appear during the anodic scans, corresponding to the successive de-lithiation reactions of Li_3_Bi (peak A) and Li_2_Te (peak B and peak C). The CV plots of bare Bi_2_Te_3_ are also given for comparison as seen in Figure 3b. The plots of bare Bi_2_Te_3 _display a similar feature as those of Bi_2_Te_3_/G except that the peak intensity decreases more rapidly with scans, indicative of its lower reversibility. The relatively high reversibility of Bi_2_Te_3_/G can be attributed to the incorporation of graphene that improves the electrode kinetics and keeps the electrode integrity.

**Figure 4:**
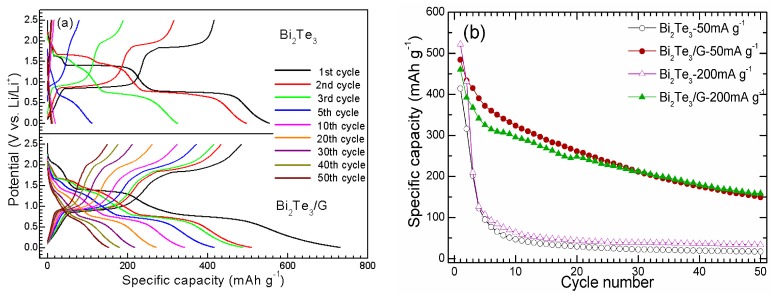
(**a**) Voltage profiles; and (**b**) cycling stability of Bi_2_Te_3_/G and Bi_2_Te_3_.

Figure 4a shows the chare-discharge curves of Bi _2_Te_3_ and Bi_2_Te_3_/G at 50 mA g^−1 ^at various cycles. Bi_2_Te_3_/G hybrid gives a first discharge (Li-uptake) capacity of 731 mAh g^−1^ and a first charge (Li-extraction) capacity of 481 mAh g^−1^. The first irreversible capacity can be attributed to the reduction decomposition of the electrolyte and the formation of the solid state interface (SEI) layer. Successive potential plateaus can be seen during the charge-discharge cycling, which correspond to the current peaks in the CV plots. The appearance of the potential plateaus is due to the two-phase coexistence of original phase (Te or Bi) and the Li-alloys (Li-Te or Li-Bi alloys). As shown in the figure, bare Bi_2_Te_3_ exhibits lower charge-discharge capacities compared with Bi_2_Te_3_/G. The theoretical maximum capacity of Bi_2_Te_3_ is 401 mAh g^−1^, related to the formation of Li_2_Te and Li_3_Bi. Considering the fact that graphene itself shows a relatively low capacity of around 300 mAh g^−1^, as reported in our previous work [33], the theoretical capacity of Bi_2_Te_3_/G should be lower than that of bare Bi_2_Te_3_. The high yieldable of Bi_2_Te_3_/G is possibly due to the synergistic effect between Bi_2_Te_3_ and graphene. On one hand, as discussed above, the presence of graphene can restrain the crystal growth of Bi_2_Te_3_, which is favorable for rapid Li ion diffusion within the active material. On the other hand, the separator effect of Bi_2_Te_3_ plates for graphene sheets maximizes the exposure of the graphene surface to the electrolyte, especially for the edges and vacancies on graphene. The edges and vacancies can supply additional sites for Li storage [34]. Note that Bi_2_Te_3_/G shows a progressive polarization upon cycling, but the extent is smaller than bare Bi_2_Te_3_ owing to the presence of the conductive graphene.

Figure 4b compares the cycling stability between bare Bi_2_Te_3_ and Bi_2_Te_3_/G charged at 50 and 200 mA g^−1^ and discharged at 50 mA g^−1^. Note that Bi_2_Te_3_/G demonstrates an obviously slower capacity fade compared to bare Bi_2_Te_3_. After 50 cycles at 200 mA g^−1^ (about 0.5 C), a charge capacity of 158 mAh g^−1^ can be retained for Bi_2_Te_3_/G. In contrast, the charge capacity of bare Bi_2_Te_3_ drops rapidly to 33 mAh g^−1^. The Bi_2_Te_3_/G hybrid also shows improved cycling stability compared with Bi_2_Te_3_-graphite composite [27] and other Bi-based anodes [25,26]. The enhanced cycling stability is attributed to the buffering effect of graphene that alleviates the large volume changes and the confinement effect of graphene that restrains the aggregation of the Bi_2_Te_3_ plates, in addition to offering effective conducting networks. The 3D sandwich structure also facilitates the Li-ion diffusion within the free space and across the electrode/electrolyte interface. In addition, the free space within the sandwich also affords additional room to buffer the volume changes. It should be stressed that the long-term cycling stability of the Bi_2_Te_3_/G hybrid is not satisfactory yet owing to the intrinsic large volume changes of the Bi_2_Te_3_ during alloying/de-alloying processes. However, it is clear that graphene plays an important role in enhancing the electrochemical properties of Bi_2_Te_3_. It also should be noted that the large-scaled of Bi_2_Te_3_/G is not favored due to the toxicity, price, and resource scarcity of Bi and Te. Nevertheless, it shows promising application in micro-batteries due to the possibility of micro processing of graphene and Bi_2_Te_3_.

## 3. Experimental Section 

### 3.1. Preparation of Bi_2_Te_3_-Nanoplate/Graphene-Nanosheet (Bi_2_Te_3_/G) Nanohybrid

Graphite oxide (GO, 80 mg), prepared by a modified Hummer’s method [35], was added into 40 mL of ethylene glycol (EG) and sonicated for 24 h to obtain a sufficiently exfoliated GO suspension. Then, 1 mmol of BiCl_3_, 1.5 mmol of Na_2_TeO_3 _and 0.4 g of NaOH was added to the above suspension with sonication. After stirring for 12 h, the mixed solution was then transferred to a Teflon-lined stainless steel autoclave (100 mL) and heated in an electric oven at 180 °C for 24 h. The as-obtained product was collected by centrifugation, washed with deionized water and absolute ethanol for several times and dried at 40 °C under vacuum for 10 h. Bare Bi_2_Te_3_ was also synthesized using a similar method without adding GO.

### 3.2. Materials Characterization

The X-ray diffraction (XRD) patterns were obtained on a Rigaku D/Max-2550pc powder diffractometer using Cu K_α_ radiation (*λ* = 0.1541 nm). X-ray photoelectron spectra (XPS) were collected on a KRATOS AXIS ULTRA-DLD spectrometer with a monochromatic Al K_α_ radiation (h*v* = 1486.6 eV). The morphologies of the products were observed by field emission scanning electron microscopy (FE-SEM) on a FEI-sirion microscope and transmission electron microscopy (TEM) on a JEM 2100F microscope. The carbon content analysis was conducted on a Flash EA 1112 tester.

### 3.3. Electrochemical Measurements

The electrochemical measurements of Bi_2_Te_3_/G and Bi_2_Te_3_ were performed with a half-cell configuration using CR2025-type coin cells. The working electrodes were prepared by spreading a slurry composed of 75 wt% active material (Bi_2_Te_3_/G or Bi_2_Te_3_/G), 15 wt% poly(vinylidene fluoride) (PVDF) and 10 wt% acetylene black onto Ni foam followed by drying at 100 °C for 12 h under vacuum. The loading mass of the active material is around 2 mg. The cells were assembled in an Ar-filled glove box using Li foil as the counter electrode and Celgard 2300 membrane as the separator. The electrolyte used was 1 M LiPF_6_ in ethylene carbonate (EC)/dimethyl carbonate (DMC) (volume ratio 1:1). The cells were galvanostatically charged-discharged at 50 mA g^−1 ^between 0.005 and 2.5 V *vs.* Li/Li^+ ^on a Neware battery tester (Shenzhen, China). The specific capacity of Bi_2_Te_3_/G was calculated based on the total weight of Bi_2_Te_3_ and graphene. Cyclic voltammetry (CV) tests were carried out on an Arbin BT2000 system at a scan rate of 0.1 mV s^−1 ^between 0.005 and 2.5 V *vs.* Li/Li^+^. All of the electrochemical measurements were carried out at room temperature.

## 4. Conclusions

The Bi_2_Te_3_/G sandwich has been prepared by an in situ route with alternatively arranged Bi_2_Te_3_ nanoplates and graphene nanosheets. The diffusion and re-crystallization of Bi_2_Te_3_ in Bi_2_Te_3_/G are inhibited due to presence of oxygen-containing groups and defects on graphene, leading to more irregular shape and smaller thickness of Bi_2_Te_3_ in Bi_2_Te_3_/G compared with those in bare Bi_2_Te_3_. Both Bi_2_Te_3_/G and Bi_2_Te_3_ exhibit a multi-step lithiation mechanism. The Bi_2_Te_3_/G hybrid can yield a higher-than-the-theoretical charge capacity of mAh h^−1^ at 50 mA g^−1^ due to the synergistic effect between Bi_2_Te_3_ and graphene. The Bi_2_Te_3_/G hybrid shows an obviously improved cycling stability compared with bare Bi_2_Te_3_. The improved cycling stability is attributed to the 3D sandwich structure, where graphene not only buffers the volume changes but also immobilizes Bi_2_Te_3_ nanoplates, in addition to offering effective conducting networks. The unique sandwich structure also facilitates Li-ion diffusion at the electrode/electrolyte interface and within the free space of the sandwich.
